# METTL3 Alleviates Liver Abscess by Enhancing Macrophage Efferocytosis via m^6^A Methylation of Nrf2 mRNA

**DOI:** 10.1002/iid3.70534

**Published:** 2026-10-06

**Authors:** Genwang Wang, Ding Luo, Yu Pang, Chanjuan Gao, Dong Jin, Qi Wang

**Affiliations:** ^1^ Department of Hepatobiliary Surgery General Hospital of Ningxia Medical University Yinchuan China; ^2^ Health Management Centre General Hospital of Ningxia Medical University Yinchuan China; ^3^ Department of Respiratory Medicine The Third People's Hospital of Yinchuan Yinchuan China

**Keywords:** efferocytosis, liver abscess, m^6^A methylation, METTL3, Nrf2

## Abstract

**Background:**

Persistent inflammation and impaired macrophage efferocytosis contribute to liver injury during bacterial liver abscesses (BLA). This study was conducted to investigate whether METTL3 alleviates BLA by promoting macrophage efferocytosis through m6A methylation of Nrf2 mRNA.

**Materials and Methods:**

METTL3 was overexpressed in the liver of a Klebsiella pneumoniae‐induced BLA mouse model. Liver injury, serum cytokine levels, Nrf2 signaling, m6A enrichment of Nrf2 mRNA, TAM receptor expression, and macrophage‐associated Mertk and Nrf2 expression were evaluated. Nrf2 knockdown rescue experiments were further performed in lipopolysaccharide‐stimulated RAW264.7 macrophages.

**Results:**

METTL3 overexpression reduced hepatic abscess formation and tissue injury, decreased serum interleukin (IL)‐1β, IL‐6, and tumor necrosis factor alpha (TNF‐α) levels, and increased IL‐10 and transforming growth factor beta (TGF‐β) levels. METTL3 overexpression also enhanced m^6^A enrichment of Nrf2 mRNA, activated Nrf2/HO‐1 signaling, and upregulated the efferocytosis‐related receptors MerTK, Tyro3, and AXL in BLA liver tissues. Immunofluorescence analysis revealed increased co‐expression of MerTK and Nrf2 in hepatic macrophages. In RAW264.7 cells, Nrf2 knockdown reversed the METTL3‐induced upregulation of MerTK, Tyro3, and AXL, as well as the decrease in CD86‐positive cells and increase in CD206‐positive cells.

**Conclusions:**

These findings demonstrate that METTL3 promotes m^6^A methylation of Nrf2 mRNA, activates the Nrf2/TAM receptor axis, enhances macrophage efferocytosis, and alleviates BLA.

## 1 Introduction

The core pathological mechanism of liver abscess (LA), a severe suppurative infectious disease, is the failure to promptly clear large amounts of necrotic cells and pathogen fragments within the infection site [1]. Consequently, this triggers an excessive and persistent inflammatory response, exacerbating local tissue damage and significantly affecting patients' clinical prognosis [2]. Without proper intervention, this may lead to peritonitis and sepsis, causing life‐threatening irreversible damage [3]. Currently, standard treatments for LA primarily include antibiotic therapy and punctaneous drainage [4]. Despite their ability to effectively eliminate pathogens and manage the abscess cavity, these strategies have limited efficacy in regulating the intrinsic capacity to mitigate inflammation [5]. Reports of poor prognosis in some patients were attributed to delayed initial treatment or an inadequate response to single‐drug therapy [6]. Therefore, an in‐depth investigation of the cellular and molecular mechanisms underlying the active resolution of inflammation in LA has clinical significance.

During LA progression, the efferocytic function of macrophages serves as a crucial regulatory hub [7]. Efferocytosis, the process by which macrophages efficiently recognize, phagocytose, and clear apoptotic and necrotic cells [8], is an essential mechanism for removing cellular debris and a crucial immunoregulatory behavior that drives macrophages toward an anti‐inflammatory and reparative phenotype. This promotes the release of relevant cellular mediators to facilitate inflammation resolution and tissue reconstruction [9, 10]. However, under high‐burden infections and stressful conditions, such as those in LA, macrophage efferocytosis is often suppressed or impaired, leading to a vicious cycle of necrotic debris accumulation, sustained inflammation, and further tissue damage [11]. In contrast, during the ideal resolution phase, effective efferocytosis constitutes the central mechanism for active resolution of inflammation and initiation of tissue repair [12]. Therefore, enhancing the efferocytic capacity of macrophages is a key scientific challenge for promoting healing of LA lesions.

Nuclear factor erythroid 2‐related factor 2 (Nrf2), a central transcription factor for cellular anti‐inflammatory defense, is critical for maintaining macrophage functional homeostasis and regulates genes related to phagocytosis [13, 14]. However, the upstream mechanisms controlling Nrf2 expression and activity in LA remain to be elucidated. Epitranscriptomic regulation, particularly N^6^‐methyladenosine (m^6^A) modification, precisely and rapidly modulates gene expression at the post‐transcriptional level, thereby playing an important role in the stress response of immune cells [15]. As the most abundant chemical modification of eukaryotic mRNA, m6A is primarily catalyzed by methyltransferases such as METTL3 and dynamically regulates target gene function by influencing mRNA stability, splicing, nuclear export, and translation efficiency [16]. Nevertheless, whether METTL3‐mediated m^6^A methylation of Nrf2 mRNA regulates Nrf2 signaling, thereby serving as a key regulatory node for macrophage efferocytosis under LA‐induced infectious stress, remains to be investigated.

This study was conducted to determine whether METTL3‐mediated m6A methylation of Nrf2 mRNA is a key mechanism for enhancing macrophage efferocytosis and promoting LA resolution in an animal model of bacterial liver abscess (BLA). Nrf2‐knockdown rescue experiments in RAW264.7 macrophages were performed to verify the causal role of Nrf2 in the METTL3‐mediated regulation of TAM receptors and macrophage polarization. The findings suggest a novel METTL3/Nrf2/efferocytosis pathway involved in inflammatory resolution during BLA.

## 2 Materials and Methods

### 2.1 Animals

A total of 40 male SPF‐grade C57BL/6 J mice (6 weeks old, 17 ± 2 g) were provided by Jiangsu Qinglongshan Biotechnology Co. Ltd. (No. SCXK (Su) 2023‐0001). All animal experiments were performed at the animal facility of Suzhou Wanwei Life Science & Technology Co. Ltd. (No. SYXK (Su) 2023‐0044). The mice were housed in a SPF barrier‐sustained environment maintained at 20°C–26°C and 40%–70% relative humidity, with a 12‐h light/dark cycle and a density of five mice per cage. All procedures involving live *Klebsiella pneumoniae* were performed under Biosafety Level 2 conditions (registration no. SZ2025241). The animal infection model was established in an Animal Biosafety Level 2 laboratory (registration No. Su Dong Shi Bei Zi [2025] No. 014).

### 2.2 Animal Experimental Design

After a 7‐day acclimatization period, the mice were randomly assigned to control, BLA model, BLA + OV‐NC, and BLA + OV‐METTL3 groups (*n* = 10). Mice in the BLA + OV‐NC and BLA + OV‐METTL3 groups received a single tail vein injection of 100 μL of adeno‐associated virus (AAV) at a dose of 1 × 10^11^ viral genomes (vg) per mouse, followed by routine maintenance for 4 weeks. The recombinant AAVs used were as follows: OV‐METTL3 (VA198‐AAV‐TBG‐MCS‐P2A‐zsGreen‐m‐*Mettl3*; titer: 9.83 × 10^12^ vg/mL; lot: V325061807; General Biol, Anhui, China) and OV‐NC (VA198‐AAV‐TBG‐MCS‐P2A‐zsGreen‐blank; titer: 7.43 × 10^12^ vg/mL; lot: V325061414; General Biol, Anhui, China). On Day 29, the mice were anesthetized by intraperitoneal injection of a mixture of 55 mg/kg Zoletil and 5 mg/kg xylazine hydrochloride. Following laparotomy, mice in all groups, except the control group, received an intrahepatic injection of 100 μL of *K. pneumoniae* suspension (1.5 × 10^8^ CFU/mL; strain TS274550 = ATCC 13883; Ningbo Mingzhou Biotechnology Co. Ltd., China). The abdomen was sutured, and the animals were monitored for 5 days until the experimental endpoint. General conditions, including food intake and respiratory rate, were observed from the start of the experiment. Body weight was measured weekly before modeling and daily following bacterial inoculation. At the end of the experiment, the mice were anesthetized with 1% sodium pentobarbital (50 mg/kg, intraperitoneally). Upon loss of consciousness and the absence of a pain response, blood samples were collected, followed by cervical dislocation and harvesting of the liver tissues.

### 2.3 Hematoxylin–Eosin (HE) Staining

Mouse liver tissue was fixed in 4% paraformaldehyde, followed by automated dehydration, embedding, and sectioning. The tissue sections were deparaffinized and rehydrated using a graded ethanol series. Subsequently, the sections were stained with hematoxylin for 5–10 min, differentiated in acidic alcohol for approximately 3 s, and blued in a weak alkaline solution. After counterstaining with alcoholic eosin for 3 min, sections were dehydrated using a graded alcohol series, cleared in xylene, and mounted with neutral resin. Images were captured under a light microscope (BA210 Digital, Motic, China).

### 2.4 Enzyme‑Linked Immunosorbent Assay (ELISA)

Blood samples were collected and incubated at room temperature for 30 min. Serum was separated by centrifugation at 1000 × *g* for 10 min. The concentrations of interleukin (IL)‐6 (No. ZC‐37988), IL‐1β (No. ZC‐37974), tumor necrosis factor alpha (TNF‐α; No. ZC‐39024), and IL‐10 (No. ZC‐37962) were quantified using commercially available ELISA kits (ZCIBIO, China), while transforming growth factor beta (TGF‐β) levels were measured using a mouse TGF‐β ELISA kit (No. HJ205; Epizyme, China) according to the manufacturers’ instructions.

### 2.5 Western Blot Analysis

Total protein was extracted from mouse liver tissue and RAW264.7 cells (No. STCC20020P, Servicebio, China) using Cell Lysis Buffer (No. P0013, Beyotime, China). After quantification and denaturation, the protein samples were separated using sodium dodecyl sulfate–polyacrylamide gel electrophoresis and transferred onto polyvinylidene difluoride membranes (No. ISEQ. 00010, Sigma‐Aldrich, USA). Membranes were blocked with 5% skim milk (No. GC310001, Servicebio) and incubated overnight at 4°C with primary antibodies against METTL3 (1:5,000; No. 15073‐1‐AP, ProteinTech, China), Nrf2 (1:1,000; No. HA721432, HUABIO, China), Keap‐1 (1:2,000, No. 10503‐2‐AP, ProteinTech), HO‐1 (1:2,000, No. HA721854; HUABIO), MerTK (1:1,000, No. abs146712; Absin, Shanghai, China), Tyro3 (1:1,000; No. A0730; Abclonal, China), AXL (1:1,000, No. R1406‐3, HUABIO), and β‐actin (1:50,000, No. AC026, Abclonal). After washing, the membranes were incubated with goat anti‐rabbit IgG (H + L) horseradish peroxidase (HRP) (1:8,000, No. AS014, Abclonal) at 37°C for 3 h. Protein bands were visualized using an automated chemiluminescence imaging system (No. 5200 Multi, Tanon, China) with an ECL (ultrasensitive) substrate (No. BL520B, Biosharp, China). β‐actin was used as the loading control to normalize target protein expression levels.

### 2.6 m^6^A RNA Immunoprecipitation (MeRIP)‐Quantitative Polymerase Chain Reaction (qPCR)

Total RNA was extracted from pulverized liver tissue using TRIzol. Following quality assessment, RNA was fragmented to approximately 100 nucleotides. For each IP reaction, RNA was incubated with IP buffer and either anti‐m6A or IgG antibody at 4°C for 2–4 h. Pre‐washed and bovine serum albumin (BSA)‐blocked Protein A/G magnetic beads were added to the mixture and incubated for 1 h at 4°C. After three washes, the bound RNA was eluted with Elution Buffer and Proteinase K at 55°C for 45 min. The eluted RNA was purified and subjected to qPCR analysis for m^6^A enrichment of Nrf2 mRNA using specific primers (Nrf2: F, 5′‐TGAACTCCTGGACGGGACTA‐3′; R, 5′‐GGCTGGGACTCGTGTTCAG‐3′; 131 bp).

### 2.7 Immunofluorescence Staining

Paraffin‐embedded liver tissue sections were deparaffinized, rehydrated, and subjected to antigen retrieval using retrieval buffer (No. GA2307051, Servicebio) in a microwave oven for 20 min. Endogenous peroxidase activity was blocked by incubation with 3% hydrogen peroxide (No. G1204, Servicebio) in the dark for 25 min. Sections were blocked with BSA (No. GC305010, Servicebio) for 30 min. For the first target, sections were incubated with the primary antibody F4/80 (1:400, No. 28463‐1‐AP, ProteinTech) overnight at 4°C, followed by an HRP‐conjugated goat anti‐rabbit secondary antibody (1:100, No. GB23303, Servicebio) for 50 min. Signal amplification was performed using TSA (FITC‐Tyramide) (1:500, No. G1222, Servicebio) and incubated for 10 min in the dark. Subsequently, a second round of antigen retrieval and serum blocking was performed. For the second target, sections were incubated with the primary antibody Mertk (1:400, No. 27900‐1‐AP, ProteinTech) overnight at 4°C, followed by an HRP‐conjugated goat anti‐rabbit secondary antibody for 30 min. Signals were developed using TSA (CY3‐Tyramide) (1:500, No. G1223, Servicebio) for 10 min in the dark. A third round of antigen retrieval and serum blocking was conducted. For the third target, the sections were incubated with the primary antibody against Nrf2 (1:100, No. ER1706‐41, HUABIO, China) overnight at 4°C, followed by an HRP‐conjugated goat anti‐rabbit secondary antibody for 30 min at 37°C. Signals were developed using TSA (CY5‐Tyramide) (1:500, No. GB27303; Servicebio) for 10 min in the dark. Finally, cell nuclei were counterstained with DAPI (No. G1012, Servicebio) for 10 min. The sections were mounted with an antifade mounting medium and imaged using a fluorescence microscope (No. VS200, Olympus, Japan). The fluorescence intensity was quantified using image analysis software (No. Halo 101‐WL‐HALO‐1, Indica Labs, USA).

### 2.8 RAW264.7 Cell Culture, Transfection, and LPS Stimulation

RAW264.7 murine macrophages were cultured in high‐glucose DMEM (No. 11965‐118; Gibco, Thermo Fisher Scientific, USA) supplemented with 10% fetal bovine serum (FBS, No. FSP500, Excellbio, China), 1% penicillin–streptomycin solution (No. P1400, Solarbio, China) at 37°C in a humidified atmosphere containing 5% CO_2_. The METTL3 overexpression plasmid (OV‐METTL3) and the corresponding empty vector (OV‐NC) were constructed by General Biology (Beijing, China). RAW264.7 cells were co‐transfected with OV‐METTL3 or OV‐NC together with Nrf2‐targeting siRNA (siNrf2) or negative‐control siRNA (siNC) using Advanced DNA/RNA Transfection Reagent (No. AD601000; Zeta Life, USA) according to the manufacturer's instructions. Cells were assigned to four groups: LPS + OV‐NC + siNC, LPS + OV‐METTL3 + siNC, LPS + OV‐NC + siNrf2, and LPS + OV‐METTL3 + siNrf2. 20 h post‐transfection, the cells were stimulated with 100 ng/mL lipopolysaccharide derived from Escherichia coli O55:B5 (LPS, No. L6529, Sigma‐Aldrich) for 24 h. Cells were harvested for western blotting, quantitative real‐time PCR, and flow cytometry.

### 2.9 Real‐Time qPCR

Total RNA was extracted from RAW264.7 cells using the MolPure Flash Cell/Tissue Total RNA Kit (No. 19221ES, Yeasen Biotechnology, China) according to the manufacturer's instructions. The extracted RNA was reverse‐transcribed into cDNA using PrimeScript RT Master Mix (Perfect Real Time, No. RR036A, Takara Bio, Japan). Real‐time qPCR was performed using TB Green Premix Ex Taq II (Tli RNaseH Plus, No. RR820A, Takara Bio). The relative mRNA expression levels of METTL3, Nrf2, MerTK, Tyro3, and Axl were calculated using the 2 − ^ΔΔ^Ct method, with *Gapdh* as the internal reference gene. The primer sequences were as follows: METTL3, F: 5′‐CAGTGCTACAGGATGACGGCTT‐3′ and R: 5′‐CCGTCCTAATGATGCGCTGCAG‐3′; Nrf2, F: 5′‐CAGCATAGAGCAGGACATGGAG‐3′; R: 5′‐GAACAGCGGTAGTATCAGCCAG‐3′; MerTK, forward 5′‐ATCATCCTCGGCTGCTTCTGTG‐3′ and R: 5′‐ACGACCAGTTGGGAATCCTCCT‐3′; Tyro3, F: 5′‐GAGGATGTCCTCATTCCAGAGC‐3′ and R: 5′‐CACTGCCACTTTCACGAAGGAG‐3′; Axl, F: 5′‐GGTGTTTGAGCCAACCGTGGAA‐3′ and R: 5′‐GCCACCTTATGCCGATCTACCA‐3′; and GAPDH, F: 5′‐CATCACTGCCACCCAGAAGACTG‐3′ and R: 5′‐ATGCCAGTGAGCTTCCCGTTCAG‐3′.

### 2.10 Flow Cytometry

Following treatment, RAW264.7 cells were collected, washed with staining buffer, and incubated with PE‐conjugated anti‐mouse CD86 (PE‐FcA98025, ProteinTech) and APC‐conjugated anti‐mouse CD206 (APC‐FcA98031, ProteinTech) for 40 min at 4°C in the dark. After washing, the cells were resuspended in phosphate‐buffered saline containing 1% BSA (1% BSA/PBS) and analyzed using a flow cytometer (BD Biosciences, USA). Compensation was established using single‐stained controls, and unstained and fluorescence‐minus‐one controls were used to define the positive gates. Data were analyzed using FlowJo (version 10.8.1, BD Life Sciences, USA). The percentages of CD86‐positive and CD206‐positive cells were used to evaluate pro‐inflammatory and anti‐inflammatory/pro‐repair macrophage phenotypes, respectively.

### 2.11 Statistical Analysis

All statistical analyses were performed using IBM SPSS Statistics version 25.0 and GraphPad Prism version 9.5. Graphs were generated using GraphPad Prism 9.5. Data are presented as mean ± standard deviation (SD). Comparisons involving one independent factor were performed using one‐way analysis of variance (ANOVA), followed by post hoc multiple comparison testing where appropriate. Data involving two independent factors were analyzed using two‐way analysis of variance (ANOVA). In contrast, longitudinal data collected from the same experimental subjects were analyzed using two‐way repeated‐measures ANOVA. *p* < 0.05 was considered statistically significant.

## 3 Results

### 3.1 METTL3 Alleviates Hepatic Tissue Damage in BLA Mice

The mice remained clinically stable throughout the experimental period. As shown in Figure 1A, compared with the control group, mice in the BLA model, BLA + OV‐NC, and BLA + OV‐METTL3 groups exhibited a significant reduction in body weight following bacterial inoculation (*p* < 0.001). Fluorescence imaging of liver sections confirmed the successful delivery of OV‐NC and OV‐METTL3 to the hepatic tissue in BLA model mice (Figure 1B). Gross examination of the liver (Figure 1C) revealed that the control group showed a bright red color with a smooth and uniform surface. In contrast, the livers from the BLA model and BLA + OV‐NC groups appeared dark red and displayed multiple focal whitish round protrusions (approximately 1–2 mm in diameter) along the lobular margins. The number of these lesions ranged from 3 to 6 and occasionally coalesced into larger areas. The lesions were noticeably firmer than those in the surrounding parenchyma. Regional yellowish abscesses presenting as irregular semi‐oval structures were observed at the edges of some hepatic lobules. The central liver surface exhibited pale‐gray or dark‐red areas. In the BLA + OV‐METTL3 group, the liver predominantly exhibited fewer focal round whitish protrusions. In addition, histopathological analysis (Figure 1D) demonstrated that compared with the control group, the BLA model group displayed mild hepatocyte necrosis accompanied by inflammatory cell infiltration, oval cell hyperplasia, and fibroblast proliferation surrounding the necrotic areas. The BLA + OV‐NC group exhibited a comparable degree of pathological changes as the BLA model group. In contrast, hepatic tissue damage was significantly attenuated in the BLA + OV‐METTL3 group compared with the BLA + OV‐NC group.

**Figure 1:**
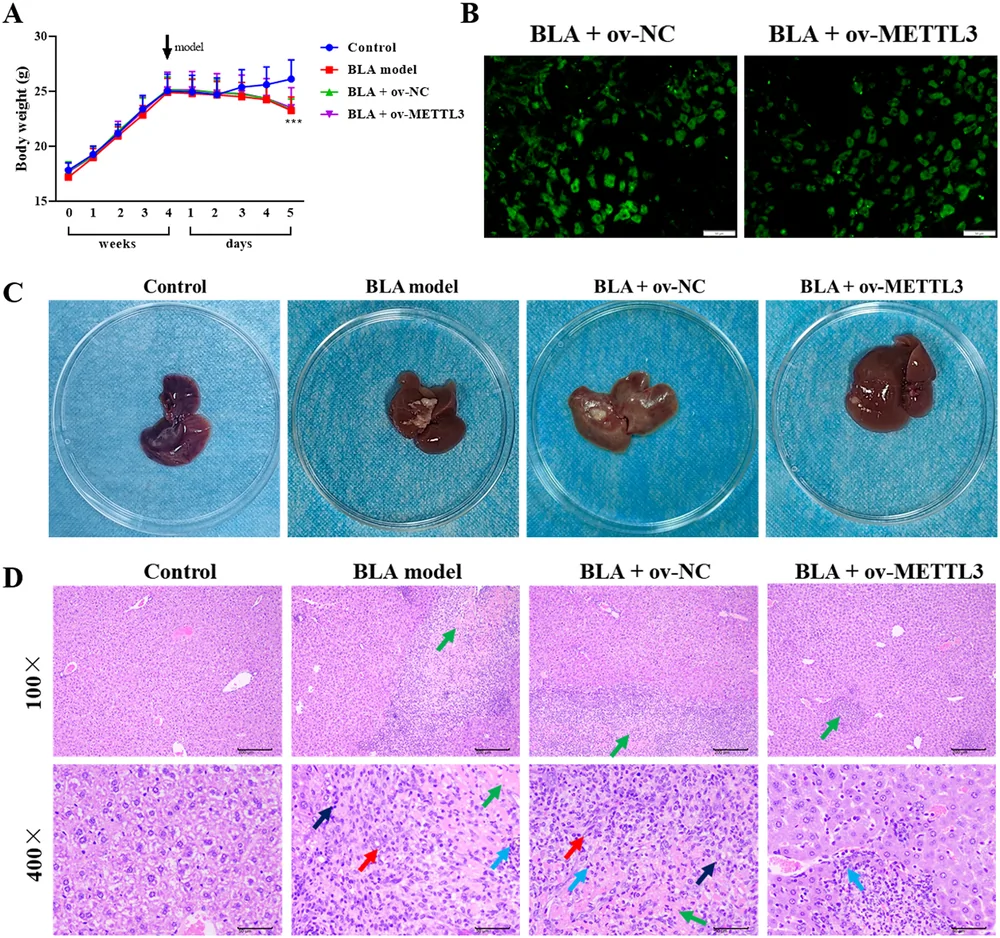
METTL3 alleviates hepatic tissue damage in BLA mice. (A) Body weight changes in mice during the experiment. (B) GFP autofluorescence in liver tissues, indicating successful plasmid delivery. (C) Representative macroscopic images of livers showing the severity of abscess formation. (D) Histopathological examination of liver sections by HE staining. Magnification: 100× and 400×. Green arrows indicate necrotic hepatocytes; blue arrows point to neutrophils; black arrows denote mononuclear cells; and red arrows highlight oval cells. ***p < 0.001, compared with the control group.

### 3.2 METTL3 Suppresses Pro‐Inflammatory Responses and Promotes Anti‐Inflammatory Cytokine Production in BLA Mice

As shown in Figure 2A,B, compared with the control group, METTL3 expression was increased in the liver tissue of the BLA model group (*p* < 0.05) and was further elevated in the BLA + OV‐METTL3 group (*p* < 0.0001), confirming successful hepatic METTL3 overexpression. Serum cytokine analysis showed that IL‐1β, IL‐6, and TNF‐α levels were significantly increased in the BLA model group compared with the control group (*p* < 0.001, Figure 2C–E). Compared with the BLA + OV‐NC group, the BLA + OV‐METTL3 group exhibited significantly lower IL‐1β, IL‐6, and TNF‐α levels and significantly higher IL‐10 and TGF‐β levels (*p* < 0.01, Figure 2F–G). These bidirectional changes in cytokine production demonstrate that METTL3 suppresses excessive inflammation and actively promotes an anti‐inflammatory and pro‐repair cytokine profile in BLA mice.

**Figure 2:**
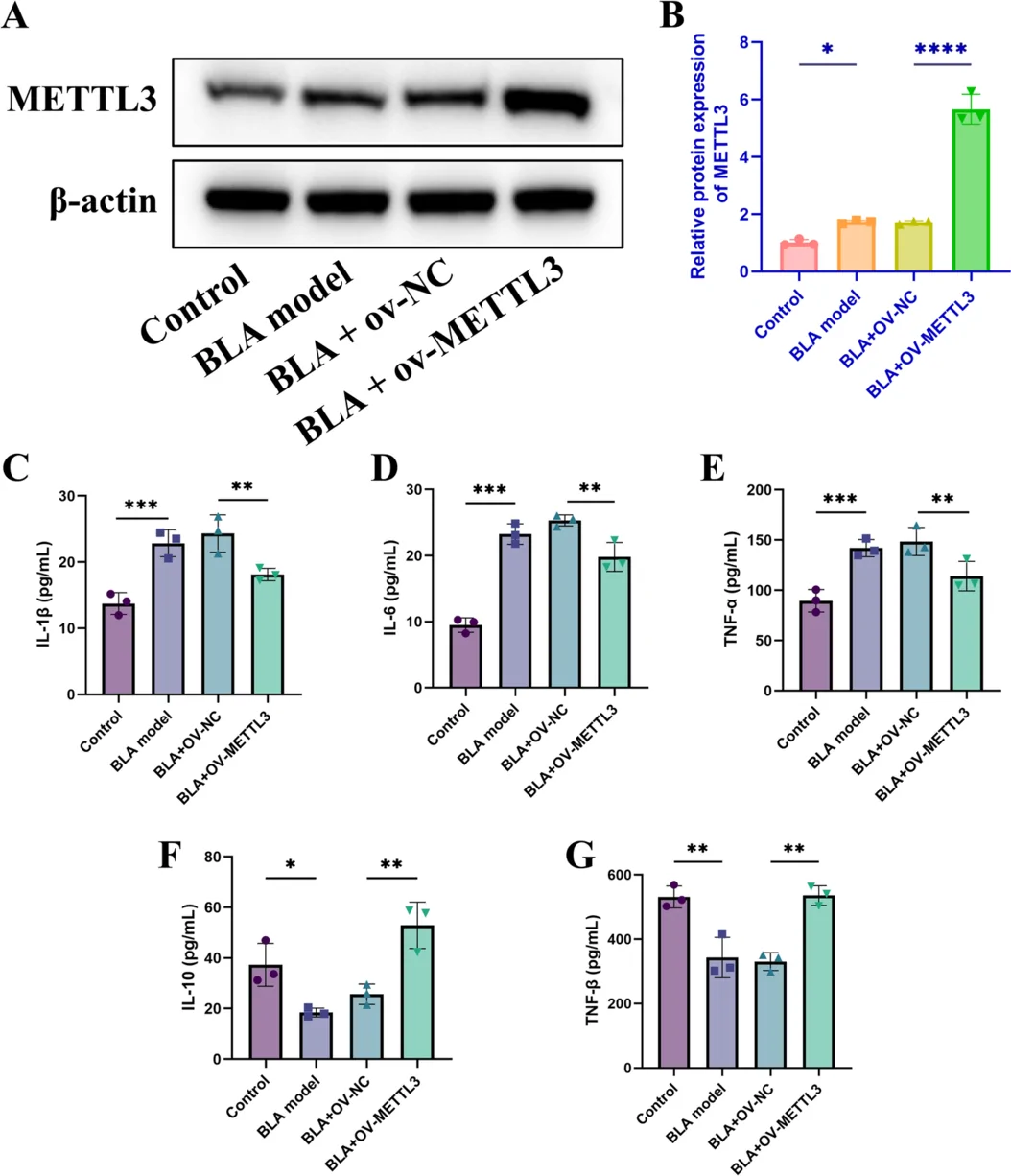
METTL3 overexpression modulates systemic inflammatory cytokine levels in BLA mice. (A) Representative Western blot images of METTL3 protein expression in liver tissues, with β‐actin used as the loading control. (B) Quantitative analysis of the relative protein expression level of METTL3. (C–G) Serum levels of IL‐1β (C), IL‐6 (D), TNF‐α (E), IL‐10 (F), and IFN‐β (G), as determined by enzyme‐linked immunosorbent assay. Data are presented as the mean ± SD (n = 3). Horizontal lines indicate the corresponding pairwise comparisons. *p < 0.05, **p < 0.01, ***p < 0.001, and ****p < 0.0001.

### 3.3 METTL3 Activates Hepatic Nrf2 Signaling and Increases m^6^A Enrichment of Nrf2 mRNA in BLA Mice

The Nrf2 signaling pathway involves a key transcription factor that inhibits excessive inflammatory responses. Western blot analysis revealed that the expression of Nrf2 and HO‐1 was significantly increased, whereas the expression of Keap‐1 was markedly decreased in the liver tissue of the BLA model group (*p* < 0.01, Figure 3A–D). These effects were enhanced in the BLA + OV‐METTL3 group, which exhibited even greater upregulation of Nrf2 and HO‐1 and stronger suppression of Keap‐1 (*p* < 0.001, Figure 3A–D). Additionally, MeRIP‐qPCR showed that m^6^A enrichment of Nrf2 mRNA was significantly increased in the liver tissue of the BLA model group and was further enhanced in the BLA + OV‐METTL3 group (*p* < 0.05, Figure 3E).

**Figure 3:**
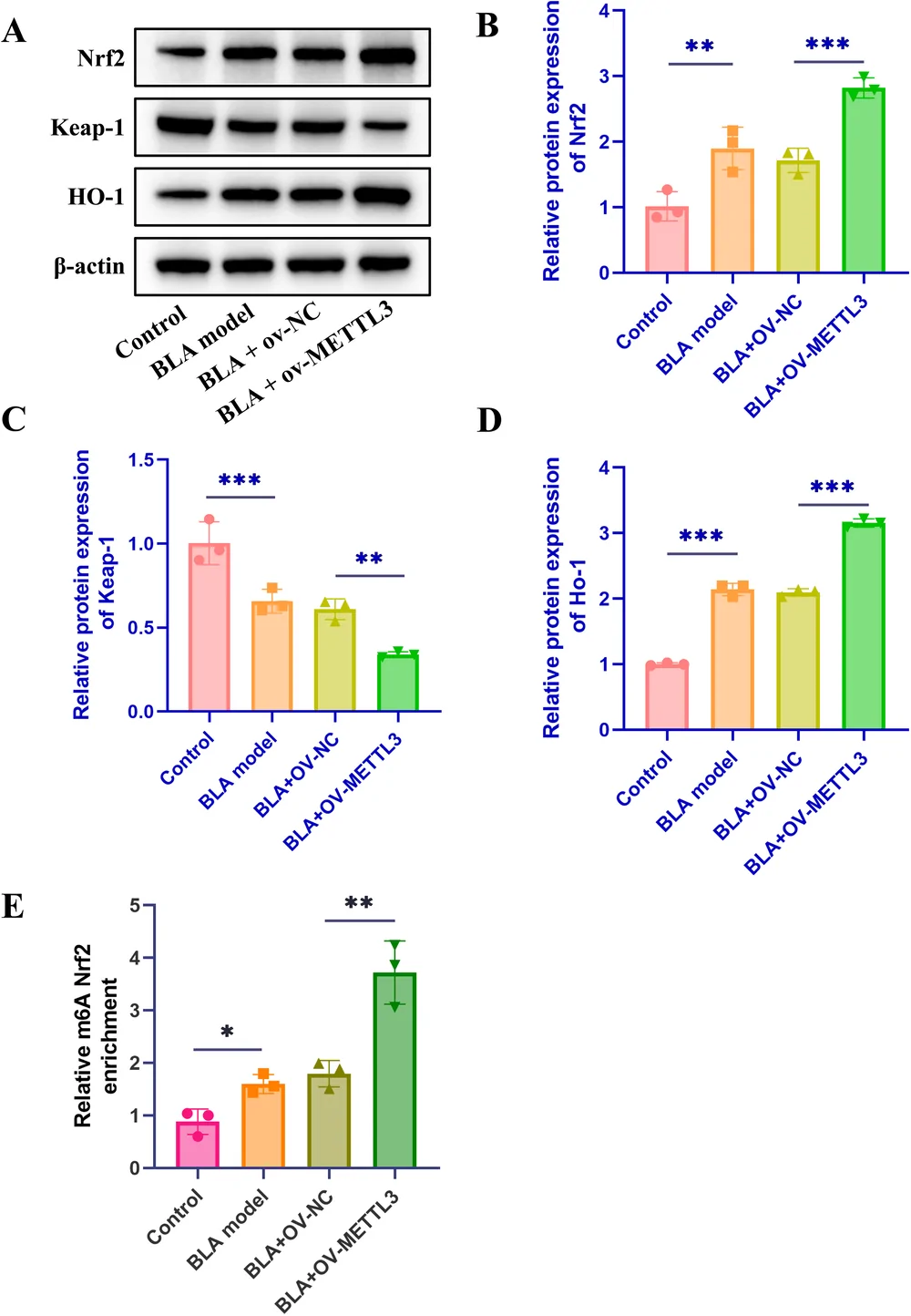
METTL3 overexpression activates the Nrf2/Keap‐1/HO‐1 signaling pathway and enhances Nrf2 mRNA m^6^A modification in the liver tissues of BLA mice. (A) Representative Western blot images of Nrf2, Keap‐1, and HO‐1 protein expression, with β‐actin used as the loading control. (B–D) Quantitative analysis of the relative protein expression levels of Nrf2 (B), Keap‐1 (C), and HO‐1 (D). (E) Relative m^6^A enrichment of Nrf2 mRNA determined by methylated RNA immunoprecipitation followed by PCR (MeRIP‐PCR). Data are presented as the mean ± SD (*n* = 3). Horizontal lines indicate the corresponding pairwise comparisons. *p < 0.05, **p < 0.01, and ***p < 0.001.

### 3.4 METTL3 Promotes Macrophage Efferocytosis and Upregulates TAM Receptors in BLA Mice

Western blotting revealed that the efferocytosis‐related TAM receptors MerTK, Tyro3, and AXL were significantly increased in the liver tissue of the BLA model group (*p* < 0.01; Figure 4), and their expression was further elevated in the BLA + OV‐METTL3 group (*p* < 0.001; Figure 4), suggesting enhanced macrophage efferocytosis. Immunofluorescence staining revealed a marked increase in F4/80‐positive macrophages co‐expressing MerTK and Nrf2 in the BLA model group (*p* < 0.05; Figure 5). This co‐expression pattern was further increased by METTL3 overexpression (*p* < 0.001, Figure 5), linking Nrf2 activation to enhanced MerTK‐associated efferocytosis in hepatic macrophages.

**Figure 4:**
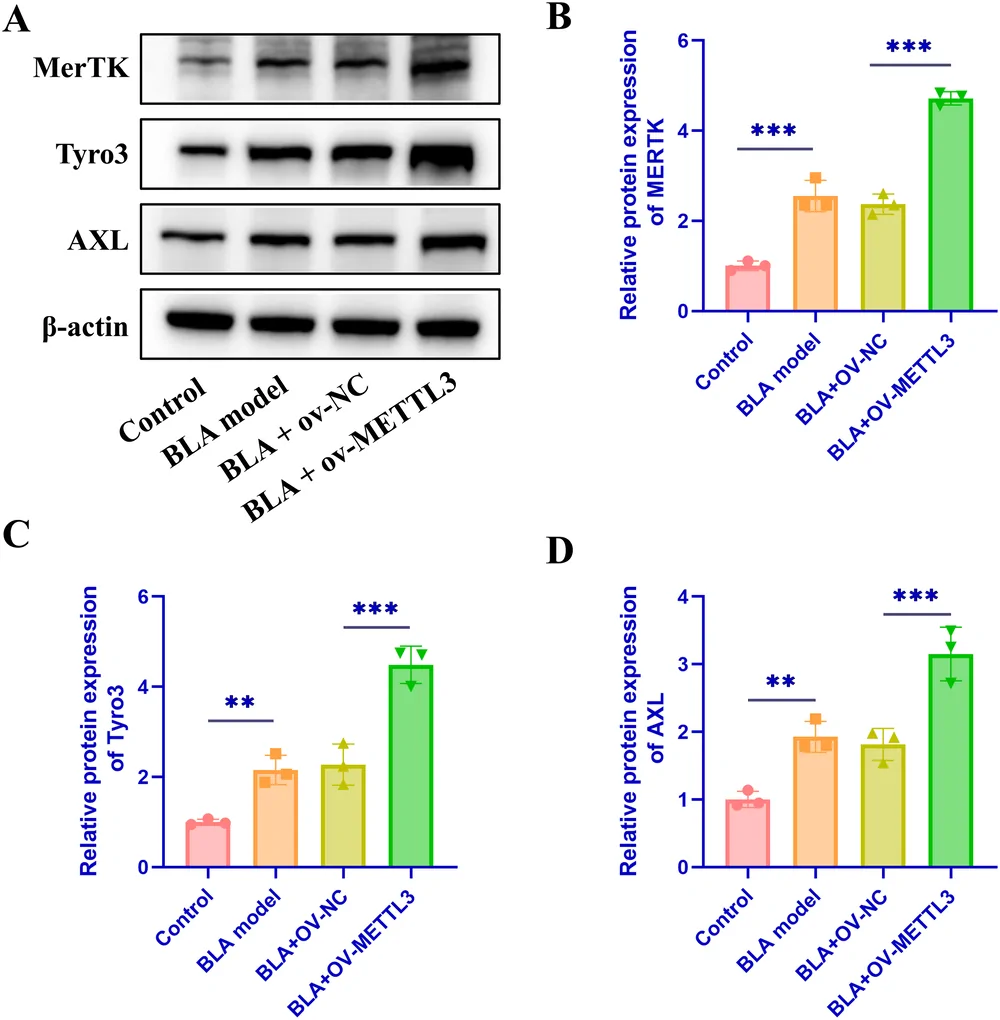
METTL3 overexpression increases the expression of efferocytosis‐related TAM receptors in the liver tissue of BLA mice. (A) Representative Western blot images of MerTK, Tyro3, and AXL protein expression, with β‐actin used as the loading control. (B–D) Quantitative analysis of the relative protein expression levels of MerTK (B), Tyro3 (C), and AXL (D). Data are presented as the mean ± SD (*n* = 3). Horizontal lines indicate the corresponding pairwise comparisons. **p < 0.01 and ***p < 0.001.

**Figure 5:**
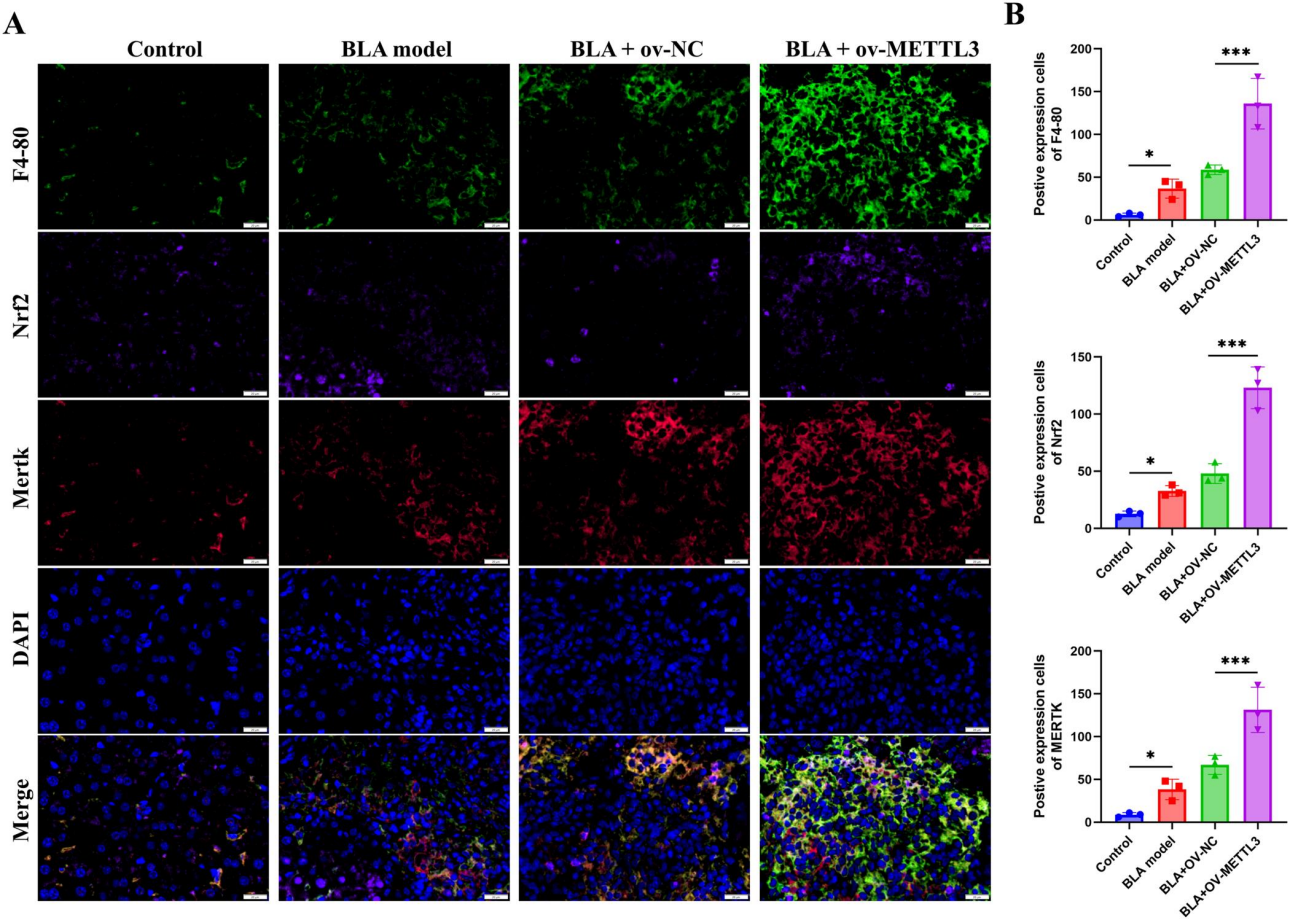
METTL3 overexpression enhances Nrf2 and MerTK expression in hepatic macrophages in BLA mice. (A) Representative immunofluorescence images of F4/80‐positive macrophages (green), Nrf2 (purple), MerTK (red), and nuclei stained with DAPI (blue), together with the corresponding merged images. Scale bars are shown in the images. (B) Quantitative analysis of the numbers of F4/80‐, Nrf2‐, and MerTK‐positive cells. Data are presented as the mean ± SD (*n* = 3). Horizontal lines indicate the corresponding pairwise comparisons. *p < 0.05 and ***p < 0.001.

### 3.5 Nrf2 Knockdown Reverses METTL3‐Induced TAM Receptor Upregulation in LPS‐Stimulated RAW264.7 Macrophages

In LPS‐stimulated RAW264.7 macrophages, OV‐METTL3 significantly increased METTL3 expression, whereas siNrf2 effectively reduced Nrf2 expression at both the mRNA and protein levels (Figure 6A–H). Compared with the LPS + OV‐NC + siNC group, METTL3 overexpression markedly increased the mRNA and protein expression of MerTK, Tyro3, and AXL (*p* < 0.01). Nrf2 knockdown reversed the increases in all three TAM receptors in the METTL3‐overexpression background (*p* < 0.01). Two‐way ANOVA revealed significant interactions between METTL3 overexpression and Nrf2 knockdown for MerTK, Tyro3, and AXL expression, demonstrating the vital role of Nrf2 in METTL3‐induced TAM receptor upregulation.

**Figure 6:**
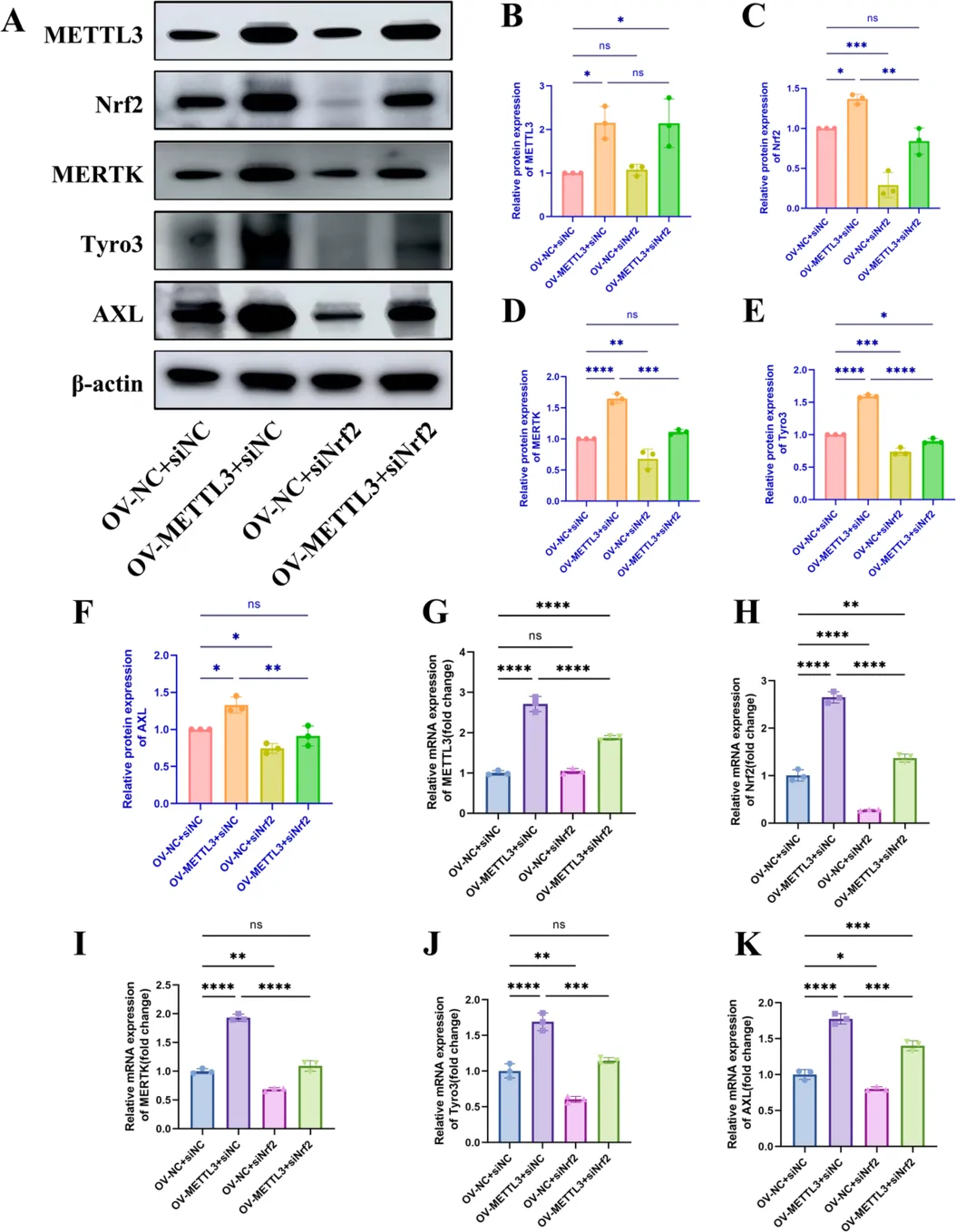
Nrf2 knockdown attenuates METTL3‐induced upregulation of efferocytosis‐related TAM receptors in LPS‐stimulated RAW264.7 macrophages. (A) Representative Western blot images of METTL3, Nrf2, MerTK, Tyro3, and AXL protein expression, with β‐actin used as the loading control. (B–F) Quantitative analysis of the relative protein expression levels of METTL3 (B), Nrf2 (C), MerTK (D), Tyro3 (E), and AXL (F). (G–K) Relative mRNA expression levels of METTL3 (G), Nrf2 (H), MERTK (I), Tyro3 (J), and AXL (K), as determined by quantitative real‐time PCR. Cells were assigned to the OV‐NC + siNC, OV‐METTL3 + siNC, OV‐NC + siNrf2, and OV‐METTL3 + siNrf2 groups. Data are presented as the mean ± SD (*n* = 3). Horizontal lines indicate the corresponding pairwise comparisons. ns, not significant; *p < 0.05, **p < 0.01, ***p < 0.001, and ****p < 0.0001.

### 3.6 Nrf2 Mediates METTL3‐Induced Anti‐Inflammatory Macrophage Polarization in LPS‐Stimulated RAW264.7 Macrophages

Flow cytometric analysis showed that, compared with the LPS + OV‐NC + siNC group, METTL3 overexpression significantly decreased the proportion of CD86‐positive macrophages (*p* < 0.05) and increased the proportion of CD206‐positive macrophages (*p* < 0.0001). In the METTL3‐overexpression background, Nrf2 knockdown significantly counteracted the reduction in CD86‐positive cells (*p* < 0.01) and attenuated the increase in CD206‐positive cells (*p* < 0.05; Figure 7A–C). These findings demonstrate that METTL3 promotes macrophage polarization toward an anti‐inflammatory and pro‐repair phenotype through Nrf2 activation.

**Figure 7:**
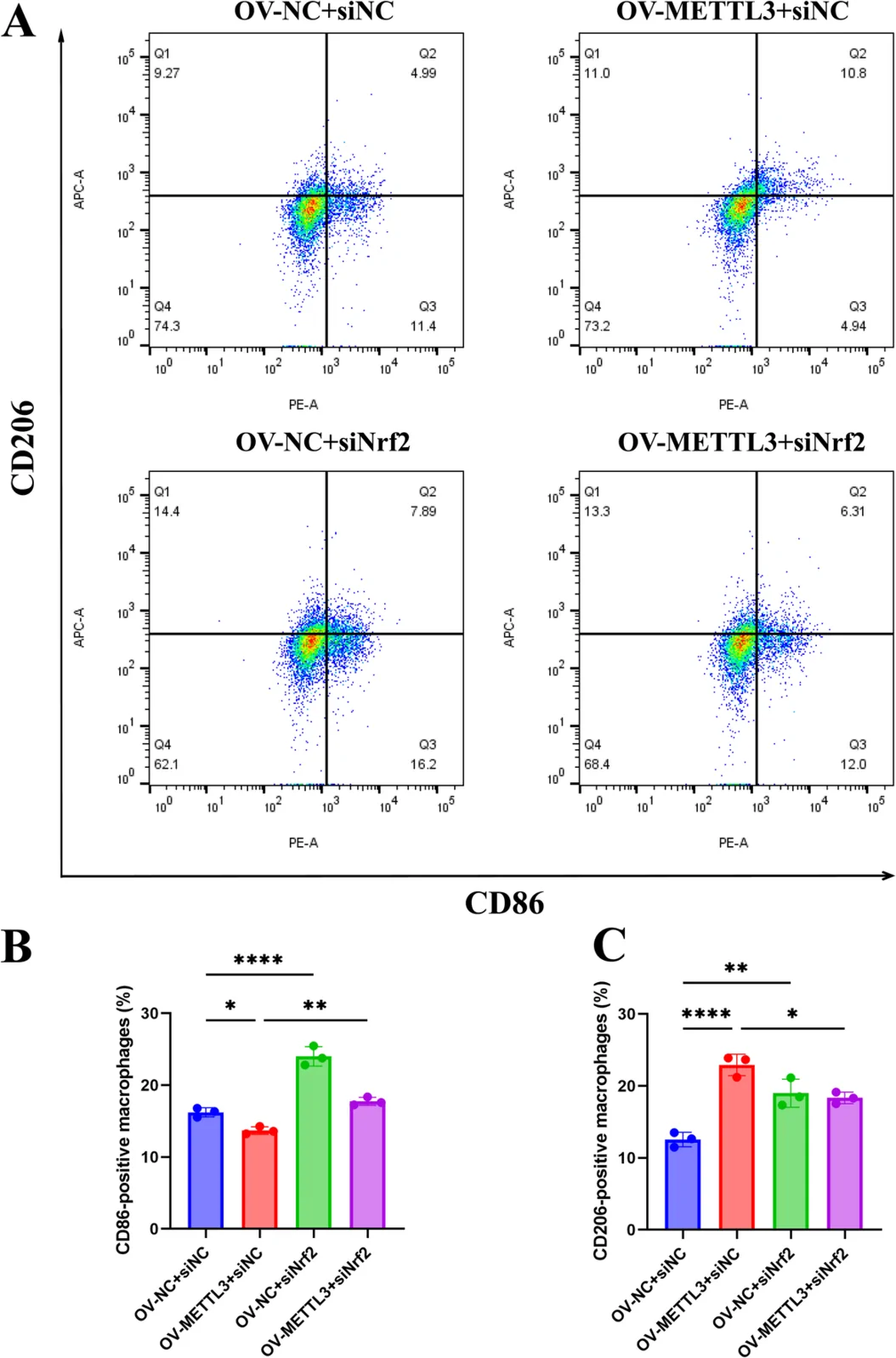
Nrf2 knockdown reverses METTL3‐induced polarization of LPS‐stimulated RAW264.7 macrophages toward an anti‐inflammatory phenotype. (A) Representative flow cytometry plots showing CD86 and CD206 expression in the OV‐NC + siNC, OV‐METTL3 + siNC, OV‐NC + siNrf2, and OV‐METTL3 + siNrf2 groups. CD86 was detected in the PE channel, and CD206 was detected in the APC channel. (B, C) Quantitative analysis of the percentages of CD86‐positive macrophages (B; Q2 + Q3) and CD206‐positive macrophages (C; Q1 + Q2). Data are presented as the mean ± SD (*n* = 3). Horizontal lines indicate the corresponding pairwise comparisons. *p < 0.05, **p < 0.01, and ****p < 0.0001.

## 4 Discussion

The pathological outcome of LA is highly dependent on timely resolution of inflammation, in which macrophage efferocytosis serves as the core regulatory step [17]. The efficiency of efferocytosis is tightly regulated at the epitranscriptomic level, with m^6^A modification serving as an important regulatory layer for inflammatory control under infectious conditions [18]. Nrf2, a key signaling hub in cellular defense against inflammation, may be regulated via METTL3‐mediated m6A methylation of Nrf2 mRNA, influencing macrophage function [19, 20]. Herein, hepatic METTL3 overexpression significantly alleviated liver tissue injury, reduced pro‐inflammatory cytokine production, increased anti‐inflammatory and pro‐repair cytokines, increased m6A enrichment of Nrf2 mRNA, activated Nrf2/HO‐1 signaling, and upregulated the TAM receptors MerTK, Tyro3, and AXL in a Klebsiella pneumoniae‐induced BLA model. In LPS‐stimulated RAW264.7 macrophages, Nrf2 knockdown reversed METTL3‐induced TAM receptor expression and macrophage phenotypic remodeling. These findings indicate that the METTL3/Nrf2 axis is a central mechanism that coordinates macrophage efferocytosis, inflammatory resolution, and tissue repair during BLA.

BLA is a severe suppurative liver disease caused by various pathogens, including bacteria, ameba, fungi, and parasites, with bacterial infections accounting for approximately 80% of cases [21]. Currently, *K. pneumoniae* is the leading pathogen responsible for LA, accounting for > 50% of the clinical isolates [22]. *K. pneumoniae* infection can trigger significant systemic and local inflammatory responses and is often accompanied by extrahepatic dissemination, complicating clinical management [23, 24]. Herein, we established a *K. pneumoniae*‐induced BLA mouse model. The significant reduction in body weight, pathological liver damage, elevated serum inflammatory cytokine levels, and activation of the Nrf2 pathway collectively validated the effectiveness of the model. Furthermore, model mice showed a marked increase in METTL3 expression, accompanied by elevated m6A methylation of Nrf2 mRNA and upregulation of key macrophage efferocytosis receptors.

m^6^A modification, the most abundant internal post‐transcriptional modification in eukaryotic mRNA, plays an important role in regulating inflammatory responses [25]. For instance, LPS alters m^6^A RNA methylation to promote excessive production of pro‐inflammatory cytokines, ultimately leading to hepatic inflammation and injury [26]. Similarly, bacterial pathogens, such as *Escherichia coli* and *Staphylococcus aureus*, induce cellular damage through mechanisms associated with m^6^A modification [27]. Herein, m6A enrichment of Nrf2 mRNA and METTL3 expression was significantly increased in the BLA model, accompanied by the activation of Nrf2 pathway proteins. Nrf2 mRNA may undergo m^6^A methylation. In various pathophysiological conditions, such as PM2.5 exposure and heavy metal‐induced injury, changes in m^6^A levels of its mRNA affect oxidative stress and inflammatory outcomes [28, 29]. Furthermore, METTL3, a key regulatory enzyme of m6A methylation, can activate the Nrf2 antioxidant pathway in a METTL3‑dependent manner [30]. METTL3 overexpression further increased the m^6^A enrichment of Nrf2 mRNA and enhanced Nrf2 pathway activation, suppressing inflammation and alleviating BLA pathology.

METTL3 expression is altered under pathological conditions, influencing and modulating the expression levels of downstream target genes and contributing to the pathogenesis of various inflammatory diseases [31]. METTL3 expression increases following myocardial infarction, promoting post‐infarction inflammation [32]. Human histological examinations have shown elevated METTL3 levels in renal tubules, whereas METTL3 silencing alleviates inflammation in renal tubular epithelial cells [33]. Similarly, METTL3 is upregulated in inflammatory bowel disease, and knockdown of METTL3 both *in vivo* and *in vitro* reduces the levels of pro‐inflammatory cytokines and inflammatory enzymes [34]. Conversely, METTL3 was downregulated in a hyperuricemic mouse model; its knockdown exacerbated neuroinflammation by enhancing hippocampal neuronal apoptosis and microglial overactivation [35]. This study observed significantly elevated METTL3 expression concurrent with increased levels of inflammatory factors in a BLA animal model, whereas its overexpression suppressed the secretion of inflammatory cytokines. These findings suggest that METTL3 is critical in BLA, potentially functioning as a compensatory protector early in the disease and exerting therapeutic anti‐inflammatory effects when overexpressed. Notably, METTL3 overexpression reduced the levels of IL‐1β, IL‐6, and TNF‐α and increased IL‐10 and TGF‐β, suggesting that METTL3 redirected the inflammatory milieu from a pro‐inflammatory state toward inflammation resolution and tissue repair rather than inducing nonspecific immunosuppression.

METTL3 is involved in regulating macrophage‐mediated inflammation and enhances m^6^A methylation of STAT1 transcripts in macrophages, promoting downstream inflammatory signaling and elevating the expression of pro‑inflammatory cytokines [36]. In addition, METTL3 is upregulated in hepatic fibrosis models and macrophages, where it exacerbates liver fibrosis by stimulating macrophage pyroptosis and inflammatory responses [37]. Herein, METTL3 overexpression significantly increased the expression of efferocytosis‐related TAM receptors MerTK, Tyro3, and AXL in BLA mouse liver tissues.

Among these, MerTK acts as a core receptor for efferocytosis, directly driving the clearance of apoptotic and necrotic cells and promoting macrophage polarization toward a reparative phenotype [38]. AXL and Tyro3 synergistically participate in fine‐tuning immune homeostasis [39]. Together, these receptors form a molecular network responsible for apoptotic cell clearance, dysfunction of which is closely associated with various pathological processes [40]. In this study, immunofluorescence co‐localization analysis revealed that the expression of both MerTK and Nrf2 synchronously increased in hepatic macrophages (F4/80^+^) following METTL3 overexpression, suggesting a potential spatial link between METTL3‑mediated Nrf2 activation and enhanced macrophage efferocytic capacity.

The link between METTL3‐mediated Nrf2 activation and TAM receptor upregulation is supported by accumulating evidence that the Nrf2/HO‐1 pathway regulates macrophage efferocytosis and TAM receptor expression. Activation of the Nrf2/HO‐1 pathway increases MerTK expression and macrophage efferocytosis, whereas Nrf2 deficiency impairs apoptotic‐cell clearance [41, 42, 43]. In microglia, Nrf2 deficiency reduces AXL and MerTK expression, and an antioxidant response element‐containing regulatory region has been identified within the AXL locus [44, 45]. Consistent with these findings, our Nrf2 knockdown experiments reversed METTL3‐induced upregulation of MerTK, Tyro3, and AXL. Nrf2 knockdown also reversed the METTL3‐induced decrease in CD86‐positive cells and increase in CD206‐positive cells, further demonstrating that Nrf2 mediates the anti‐inflammatory and pro‐repair effects of METTL3‐driven macrophage polarization. Together, these results demonstrate that METTL3 activates an Nrf2‐dependent TAM receptor program, promoting an anti‐inflammatory, pro‐repair, and pro‐efferocytic macrophage phenotype. Tyro3 may be incorporated into this regulatory network via indirect transcriptional or m6A‐dependent mechanisms.

This study has some limitations. TBG‐driven AAV induced hepatic rather than macrophage‐specific METTL3 overexpression, and Nrf2 rescue experiments were performed in RAW264.7 cells rather than in primary Kupffer cells or macrophage‐specific genetic models. Additionally, direct apoptotic cell engulfment and Nrf2 binding to the MerTK, Tyro3, and AXL promoter regions were not examined, and the study was limited to a single bacterial strain and one terminal time point. Therefore, future studies should employ macrophage‐specific genetic models and primary hepatic macrophages together with direct efferocytosis assays, ChIP‐qPCR or CUT& Tag, promoter–reporter analyses, and mapping of m6A sites within Nrf2 mRNA and corresponding reader proteins to further define the cell‐specific and molecular mechanisms of the METTL3/Nrf2 axis.

## 5 Conclusion

The results of this study demonstrated that METTL3 alleviates Klebsiella pneumoniae‐induced BLA by promoting m^6^A methylation of Nrf2 mRNA and activating Nrf2/HO‐1 signaling. Nrf2 activation upregulates the TAM receptors MerTK, Tyro3, and AXL and promotes macrophage polarization toward an anti‐inflammatory and pro‐repair phenotype, enhancing efferocytosis and accelerating inflammatory resolution. The reversal of these molecular and phenotypic changes by Nrf2 knockdown in RAW264.7 macrophages confirms Nrf2 as a key downstream mediator of METTL3 and establishes the METTL3/Nrf2 axis as an important regulatory pathway in the BLA.

## Author Contributions

**Genwang Wang:** writing – original draft, data curation, validation, visualization, formal analysis. **Ding Luo:** data curation, writing – original draft, methodology. **Yu Pang:** software, writing – review and editing. **Chanjuan Gao:** software, writing – review and editing. **Dong Jin:** software, methodology, project administration, supervision, resources, funding acquisition. **Qi Wang:** methodology, software, project administration, supervision, resources, funding acquisition.

## Conflicts of Interest

The authors declare no conflicts of interest.

## Declaration of AI and AI‐assisted Technologies in the Writing Process

During the preparation of this work the authors used DeepSeek in order to check spell and grammar. After using this tool, the authors reviewed and edited the content as needed and takes full responsibility for the content of the publication.

## Data Availability

The datasets used and/or analyzed during the current study are available from the corresponding author on reasonable request. This protocol was reviewed and approved by the Medical Research Ethics Review Committee of the General Hospital of Ningxia Medical University (Approval No. KYLL‐2023‐0207).
